# Interactive effects of landscape history and current management on dispersal trait diversity in grassland plant communities

**DOI:** 10.1111/1365-2745.12199

**Published:** 2013-12-18

**Authors:** Oliver Purschke, Martin T Sykes, Peter Poschlod, Stefan G Michalski, Christine Römermann, Walter Durka, Ingolf Kühn, Honor C Prentice

**Affiliations:** 1 Department of Physical Geography and Ecosystem Science, Lund UniversitySölvegatan 12, SE-223 62, Lund, Sweden; 2 Biodiversity, Department of Biology, Lund UniversitySölvegatan 37, SE-223 62, Lund, Sweden; 3 German Centre for Integrative Biodiversity Research (iDiv) Halle-Jena-LeipzigDeutscher Platz 5e, DE-04103, Leipzig, Germany; 4 Geobotany and Botanical Garden, Institute of Biology, Martin Luther University of Halle-WittenbergAm Kirchtor 1, DE-06108, Halle (Saale), Germany; 5 Institute of Botany, Faculty of Biology, University of RegensburgUniversitätsstrasse 31, DE-93053, Regensburg, Germany; 6 Department of Community Ecology, Helmholtz Centre for Environmental Research – UFZTheodor-Lieser-Strasse 4, DE-06120, Halle (Saale), Germany; 7 Plant Biodiversity, Institute of Systematic Botany, Friedrich Schiller University JenaPhilosophenweg 16, DE-07743, Jena, Germany

**Keywords:** community assembly, determinants of plant community diversity and structure, functional divergence, functional richness, historical anthropogenic impacts, landscape fragmentation, persistence, phylogenetic autocorrelation, seminatural grasslands, spatial autocorrelation

## Abstract

1. Plant communities and their ecosystem functions are expected to be more resilient to future habitat fragmentation and deterioration if the species comprising the communities have a wide range of dispersal and persistence strategies. However, the extent to which the diversity of dispersal and persistence traits in plant communities is determined by the current and historical characteristics of sites and their surrounding landscape has yet to be explored.
2. Using quantitative information on long-distance seed dispersal potential by wind and animals (dispersal in space) and on species' persistence/longevity (dispersal in time), we (i) compared levels of dispersal and persistence trait diversity (functional richness, FRic, and functional divergence, FDiv) in seminatural grassland plant communities with those expected by chance, and (ii) quantified the extent to which trait diversity was explained by current and historical landscape structure and local management history – taking into account spatial and phylogenetic autocorrel.
3. Null model analysis revealed that more grassland communities than expected had a level of trait diversity that was lower or higher than predicted, given the level of species richness. Both the range (FRic) and divergence (FDiv) of dispersal and persistence trait values increased with grassland age. FDiv was mainly explained by the interaction between current grazing intensity and the amount of grassland habitat in the surrounding landscape in 1938.
4. *Synthesis*. The study suggests that the variability of dispersal and persistence traits in grassland plant communities is driven by deterministic assembly processes, with both history and current management (and their interactions), playing a major role as determinants of trait diversity. While a long continuity of grazing management is likely to have promoted the diversity of dispersal and persistence traits in present-day grasslands, communities in sites that are well grazed at the present day, and were also surrounded by large amounts of grassland in the past, showed the highest diversity of dispersal and persistence strategies. Our results indicate that the historical context of a site within a landscape will influence the extent to which current grazing management is able to maintain a diversity of dispersal and persistence strategies and buffer communities (and their associated functions) against continuing habitat fragmentation.

## Introduction

Empirical and theoretical studies suggest that the current and ongoing loss of biodiversity is likely to have negative effects on ecosystem functioning and stability (Chapin *et al.* 2000; Loreau *et al.* 2001; Hooper *et al.* 2005; Isbell *et al.* 2011; Cadotte 2013) and that it is the functional component of biodiversity (variability in species' traits) rather than species' taxonomic identities that will determine how ecological communities respond to environmental change (Lavorel & Garnier 2002; Laliberté *et al*. 2010).

Land use change, habitat fragmentation and habitat deterioration are major threats to plant biodiversity at both global and local scales (Vitousek *et al.* 1997; Sala *et al.* 2000; Foley *et al.* 2005). The ways in which plant communities, and their associated functions, are able to track suitable habitat will be largely determined by the dispersal and persistence traits of their component species. Plants have multiple strategies for dispersal in space (e.g. long-distance dispersal by wind and animals) and time (e.g. persistence in the seed bank or as long-lived perennials), and there is also substantial interspecific variation in dispersal potential for each of these strategies (Poschlod, Tackenberg & Bonn 2005; Ozinga *et al.* 2009; Purschke *et al.* 2013). If the species within a local community represent a wide variety of dispersal and persistence strategies, the loss, for example, of a specific dispersal vector may be compensated for – if alternative dispersal mechanisms allow for successful colonization. The diversity of dispersal and persistence traits within plant communities therefore represents an important facet of biodiversity that is expected to determine how communities, and their associated functions, are sustained under future habitat fragmentation and changes in local management (see Mayfield, Ackerly & Daily 2006).

Previous studies of dispersal trait diversity have focussed on the number of dispersal syndromes and do not allow for interspecific variation in dispersal traits or the fact that dispersal potential is multidimensional (e.g. Ozinga *et al.* 2004; Mayfield, Ackerly & Daily 2006). There is therefore a need for studies that assess multi-trait dispersal potential (especially in fragmented landscapes) and its response to environmental drivers (McGill *et al.* 2006; Villéger, Mason & Mouillot 2008; Mouchet *et al.* 2010).

The variation in dispersal and persistence traits among the co-occurring species within a community is constrained by a set of nested (hierarchical) filtering processes that act over a range of spatial scales (Keddy 1992; Zobel 1997; Lebrija-Trejos *et al.* 2010; Algar, Kerr & Currie 2011). At the landscape scale, spatial isolation resulting, for example, from low amounts of suitable habitat in the surrounding landscape acts as a filter that decreases rates of long-distance dispersal and colonization success (Eriksson, Cousins & Bruun 2002), and may only allow a subset of species with a specific suite of dispersal traits to coexist. At the local scale, a lack of suitable microsites may reduce the probability of successful colonization by seed and favour long-term persistent species (Grubb 1977; Bullock *et al.* 1995). Both local and landscape filters may act in concert (Bullock *et al.* 2002; Purschke *et al.* 2012) and generate communities that are characterized by a lower variety of different dispersal and persistence strategies than would be expected from a random draw of species from the regional species pool (trait convergence). However, if, for example, both landscape structure and the availability of gaps for establishment favour multiple alternative dispersal strategies, local communities will consist of species that have a wide variety of complementary dispersal and persistence strategies (*cf*. Grime 2006; Schleicher, Peppler-Lisbach & Kleyer 2011) and the observed diversity of dispersal and persistence traits will be higher than expected (trait overdispersion).

Dispersal filtering may also have a historical component. Dispersal limitation and long-term persistence have been shown to lead to a time-lag in species' responses to habitat fragmentation and changes in local management regime (Helm, Hanski & Pärtel 2006; Herben *et al.* 2006). Previous studies have drawn attention to the importance of history as a determinant of dispersal potential in present-day plant communities and have shown that the distribution of individual dispersal and persistence traits may be related to past rather than to present-day descriptors of sites and their surrounding landscape (Adriaens, Honnay & Hermy 2006; Lindborg 2007; Purschke *et al.* 2012). However, while previous studies on the linkages between landscape history and dispersal potential have focussed on single traits, impacts of past anthropogenic activity on the diversity of dispersal and persistence strategies within present-day communities have yet to be explored.

Analyses of the relationships between trait-based indices (such as trait diversity) and environmental variables are often limited by the presence of spatial and/or phylogenetic autocorrelation, which may introduce bias in the estimation of model coefficients. It has recently been recognized that spatial structure and phylogenetic information should be considered jointly: trait-similarity between species may be the result of a shared evolutionary history, and traits may also show recent convergence as a result of adaptation to similar environmental conditions in spatially adjacent sites (Diniz-Filho *et al.* 2007; Freckleton & Jetz 2009; Kühn, Nobis & Durka 2009).

The main aim of the present study was to assess the extent to which the diversity of dispersal and persistence traits in present-day seminatural grassland plant communities is determined by the current and historical characteristics of the local management regime and the configuration of the surrounding landscape. Seminatural grasslands are among the most diverse habitats in Europe (Poschlod & WallisDeVries 2002; WallisDeVries, Poschlod & Willems 2002), and long-distance dispersal by multiple vectors has been shown to be of central importance for the colonization and maintenance of species diversity in these plant communities (Fischer, Poschlod & Beinlich 1996; Tackenberg, Poschlod & Bonn 2003). However, the substantial reduction in the area of seminatural grasslands over the last few centuries has led to a decline in the contribution of dispersal processes to colonization success in the present-day landscape (Poschlod & Bonn 1998; Schupp, Jordano & Gómez 2010; Purschke *et al.* 2012).

The first objective of our study was to quantify the extent to which levels of dispersal and persistence trait diversity [multi-trait functional richness (FRic) and functional divergence (FDiv)] within the present-day grassland plant communities were higher or lower than expected from a random draw of species from the regional species pool – taking into account five quantitative dispersal and persistence traits. If there are dominant filtering processes that either restrict the distribution of traits or that select for alternative/complementary dispersal strategies, we expect that the observed functional diversity will, on average, be either less or greater than predicted.

The second objective of the study was to quantify (taking into account both spatial and phylogenetic autocorrelation) the extent to which the configuration of the present-day and historical landscapes, the current management status and the history of management, as well as interactions between these factors, may act as filters that constrain dispersal trait diversity within present-day communities. If historical landscape configurations and management regimes have facilitated dispersal (by multiple processes) and establishment, and if there is a time-lag in species' responses to environmental change, we expect that the diversity of dispersal and persistence strategies will be explained by past, rather than current, characteristics of the grassland sites and their surrounding landscape. If the effect of local management on dispersal and persistence trait diversity is determined by the characteristics of the landscape surrounding that site (at the present day or in the past), trait diversity should be explained by an interaction between local and landscape descriptors.

## Materials and methods

### Study Area

The study area is situated on the Baltic Island of Öland and covers an area of approximately 22 km^2^. The landscape has an overall flat topography and consists of a mosaic of grassland, arable fields and forests. The proportion of seminatural grassland in the landscape has declined progressively over the last three centuries, from 86% in 1723 to 9% at the present day (Johansson *et al.* 2008).

### Vegetation Sampling

The presence–absence of herbaceous, vascular plant species was recorded between May and August 2007 in 113 grassland polygons (sites) that were classified according to their grassland continuity (age), previous land use (old grasslands or arable fields), tree cover and moisture status. Each grassland polygon represents a spatially delimited area that is relatively homogeneous in terms of tree cover and moisture status, and belongs to a single category of grassland continuity and single type of previous land use (Johansson *et al.* 2008). Vegetation sampling was restricted to dry grassland vegetation with low levels of eutrophication, in order to avoid major gradients of edaphic variation. A previous study by Reitalu *et al*. (2012) in the study area, using the same standardized vegetation sampling strategy, found low between-plot variation in soil characteristics (water content, organic matter content, pH, total nitrogen and total phosphorus content, and plant-available phosphorus) and revealed no significant relationship between edaphic variation and species richness. In each grassland polygon, we searched for all herbaceous vascular plant species within vegetation that contained the grasses *Festuca ovina* and/or *Helictotrichon pratense*. Both of these species are widespread in mesic and dry grasslands in the study area and avoid eutrophied habitats (Prentice *et al.* 2007). To reduce edge effects (see Reitalu *et al.* 2008), we did not sample the area within a 2-m zone along the polygon border. Sampling time was proportional to the polygon area and ranged between 1 and 12 h. A total of 185 species was recorded in the 113 polygons.

### Local and Landscape Descriptors

Each grassland polygon was assigned to one of four age classes (Age), corresponding, respectively, to 30, 55, 105 and 275 years of grassland continuity before 2004 (Johansson *et al.* 2008). Present-day grazing intensity (Grazing) was estimated on a scale of 0–4 (ungrazed to heavily grazed) on the basis of the presence of grazing animals and signs of recent grazing (see Reitalu *et al.* 2008). We also quantified the cover of trees (Tree.cov, in %), as descriptor for light availability (shading) and litter accumulation (Reitalu *et al.* 2008), and the total area (Area, in ha) for each grassland polygon. The percentage of seminatural grassland habitat within the present-day and historical landscape (Grass.1835, Grass.1938 and Grass.2004) within a 300-m zone around the edge of each of the grassland polygons was quantified by Johansson *et al*. (2008), using historical maps from three different time periods: 1835, 1938 and 2004.

### Dispersal and Persistence Traits

Quantitative information on five life-history traits related to long-distance seed dispersal and persistence was compiled from large trait data bases for the North-West European flora (Poschlod *et al.* 2003; Kleyer *et al.* 2008).

Long-distance dispersal potential was characterized by (i) wind dispersal potential (Wind), ranging from 0 (low) to 7 (high) on an ordinal scale, derived from data on seed terminal velocity and seed release height (Tackenberg 2003); (ii) epizoochory potential (Epizoo); and (iii) endozoochorous dispersal potential (Endozoo). Because cattle are the main type of grazing livestock in the study area, epizoochory potential, that is, cattle-coat seed retention potential, was predicted from seed mass and seed morphology using the regression model proposed by Römermann, Tackenberg & Poschlod (2005). Endozoochorous dispersal potential (Endozoo) was estimated on a continuous scale, according to the approach of Bruun & Poschlod (2006), as the number of germinated seeds from cattle dung samples corrected by the seed production per unit area (see also Purschke *et al.* 2012).

Persistence was characterized by (i) adult plant longevity (Longev), derived from data on plant life span and on clonal propagation, using three ordinal classes ‘annual and biennial’, ‘perennial/without the ability to spread clonally’, and ‘perennial showing clonality’; and (ii) seed bank persistence (SBank), based on the longevity index (Bekker *et al.* 1998), which represents the proportion of non-transient seed bank records in the data base of Thompson, Bakker & Bekker (1997).

The following species, or groups of species, could not be unequivocally distinguished on the basis of vegetative material: *Allium oleraceum/vineale, Carex caryophyllea/ericetorum*, *Cerastium glutinosum/pumilum, Fragaria vesca/viridis, Myosotis stricta/ramosissima, Polygala comosa/vulgaris*, *Prunella grandiflora/vulgaris*, *Trifolium campestre/dubium*, *Alchemilla* spp. *Corydalis* spp., *Melampyrum* spp. Trait data were pooled for the species (which are closely related and have similar trait values) within these groups.

### Trait Diversity Indices

For each grassland site, multivariate trait diversity (including all five dispersal and persistence traits) was characterized by two, complementary, indices of diversity: FRic and FDiv, according to the framework of Villéger, Mason & Mouillot (2008). FRic is a measure of the multivariate range of trait values, or the functional space, occupied by species in the community. FDiv measures how species are distributed within this functional space and thus the degree to which species cluster at the edges of the trait space. Low FDiv values indicate that most species cluster around the centre of the multivariate trait space, whereas high values indicate the predominance of species with extreme trait values that lie in the edges of the trait space. We did not estimate the third functional diversity component of Villéger, Mason & Mouillot (2008), functional evenness, because it performs poorly with presence–absence data (Mouchet *et al.* 2010; Mason *et al.* 2013). Calculations of FRic and FDiv were based on the set of 143 species (78% of the total number of species recorded in the sites) that were represented by data on at least three of the five traits. Because our study included both continuous and ordinal traits, and because trait data were not available for all species, FRic and FDiv were calculated according to the distance-based generalization of the original approach by Villéger, Mason & Mouillot (2008), as implemented in the package ‘FD’ (Laliberté & Legendre 2010) in the R statistical package (R Development Core Team 2013). We used dimensionality reduction, based on principal coordinates analysis (PCoA), and the first 15 principal coordinates (explaining 30.3% of the total trait variation) were used in the calculation of FRic and FDiv instead of the actual trait values. The PCoA was based on a Gower-distance matrix obtained from the standardized trait data.

### Analysis

#### Null model analysis

We carried out null model analysis, to test whether the observed trait diversity values (FRic and FDiv) simply reflected levels of species richness, or whether there were underlying trait-based filtering mechanisms that caused the observed values of functional diversity to be higher or lower than expected from a random draw of species from the species pool (Mason *et al.* 2007). Random communities were generated using the trial swap algorithm (Miklos & Podani 2004) in the R-package ‘vegan’ (Oksanen *et al.* 2013); swapping species occurrences among the grassland sites but keeping both the species richness at each site and the occurrence frequency of each species across the whole landscape constant. This null model takes into account the fact that (i) the number of species in a grassland site will constrain the range of possible trait values and (ii) the species are dispersal-limited and the ability to colonize a grassland site will depend on a species' frequency in the study area. For each site, the trait diversity indices were recalculated for 999 randomizations to test whether the observed trait diversity values were significantly (*P *<* *0.05) higher or lower than expected by chance. We calculated the standardized effect size (SES) according to Gotelli & Rohde (2002) as the ratio between observed to expected values of trait diversity: SES = (Obs − Exp)/sd(Exp), where Obs is the observed trait diversity value and Exp and sd(Exp) are the mean and the standard deviation of the expected trait diversity in the 999 random communities. The SES is independent of species richness (in our study: FRic: *r *=* *0.08, n.s.; FDiv: *r *=* *−0.12, n.s.), and negative or positive SES values indicate that species in a local community are more similar or dissimilar, with regard to their dispersal and persistence trait values, than predicted by chance.

We tested whether the mean SES of the grassland sites differed from zero (one-sample *t-*test), to assess whether the trait diversity of the grassland sites was, on average, lower or higher than random expectations. The average functional diversity of the sites is assumed to be random if approximately 95% of the SES values fall within the range between −2 and 2 (Gotelli & Rohde 2002; see also Kembel & Hubbell 2006). We also tested whether the number of grassland sites that had significantly higher or lower trait diversity values than expected (from the 999 random communities) was greater than expected, using a one-tailed binomial test. SES values, instead of the observed FRic and FDiv values, were used in all the subsequent analyses.

We used principal components analysis (PCA) to visually inspect to what extent the multivariate range or divergence of traits (FRic or FDiv) were related to the mean values (or to the range and divergence) of particular traits. FDiv for single traits was calculated according to Mason *et al*. (2003). PCA was carried out on the community-level mean trait values, or the range and divergence of single traits, and the multivariate FRic- and FDiv-vectors were projected onto the trait means, ranges and divergences using the *envfit*-function in ‘vegan’ (Oksanen *et al.* 2013).

#### Drivers of diversity in dispersal and persistence traits

We used GLM regression analyses to quantify and test the relationships between dispersal/persistence trait diversity (SES of FRic and FDiv) and the historical and current descriptors of the grassland sites and their surrounding landscape. All explanatory variables were scaled to mean = 0 and SD = 1 prior to analysis. Absolute Pearson correlation coefficients, |*r*|, between explanatory variables did not exceed 0.3 (see Table S1 in Supporting Information). In order to test for possible nonlinear effects (Reitalu *et al.* 2010; Pakeman 2011), as well as the possibility that the effect of local management on trait diversity may depend on landscape context (Rundlöf & Smith 2006), we ran a series of separate models to select significant quadratic effects, and significant two-way interactions between the local and landscape descriptors. To obtain the minimal adequate model that best described the data, we then carried out a stepwise backward variable selection procedure on the full model, including all linear effects as well as the pre-selected significant quadratic effects and two-way interactions from the *a priori* selection. Quadratic effects in the final, reduced model were only reported if the lowest or highest value of the quadratic curve was within the range of values for a particular explanatory variable [tested using the Mitchell-Olds & Shaw (1987) test in ‘vegan’ (Oksanen *et al.* 2013)].

We checked and, if necessary, corrected for spatio phylogenetic autocorrelation in the residuals of the minimal adequate model using the spatio-phylogenetic eigenvector filtering approach proposed by Kühn, Nobis & Durka (2009). First, the phylogenetic distance between the sites (phylogenetic beta diversity) was assessed using the 1-phylosor index (Bryant *et al.* 2008), R-package ‘picante’ (Kembel *et al.* 2010), which is defined by the fraction of branch length, in a phylogenetic tree, shared between two communities. A phylogenetic tree for the 143 species in our study was extracted from a dated, ultrametric supertree for Central European vascular plant species (Daphne 1.0, Durka & Michalski 2012; Fig. S1). Secondly, the phylogenetic distance matrix was decomposed into its eigenvectors using PCoA in the R-package ‘ape’ (Paradis, Claude & Strimmer 2004). The set of 47 eigenvectors that corresponded to positive eigenvalues were used as predictors for phylogenetic information structured at different spatial scales (i.e. spatio-phylogenetic autocorrelation). Finally, we selected those eigenvectors (Fig. S2) that reduced residual autocorrelation in the minimal adequate non-spatial models below a significance level of alpha = 0.05, using Moran eigenvector filtering (Dray, Legendre & Peres-Neto 2006) and the ME-function (modified by I. Kühn) in the R-package ‘spdep’ (Bivand *et al.* 2013). The selected eigenvectors were included as co-variables in the minimal adequate regression models in order to correct for spatio-phylogenetic autocorrelation.

## Results

### Null Model Analysis

**Fig 1:**
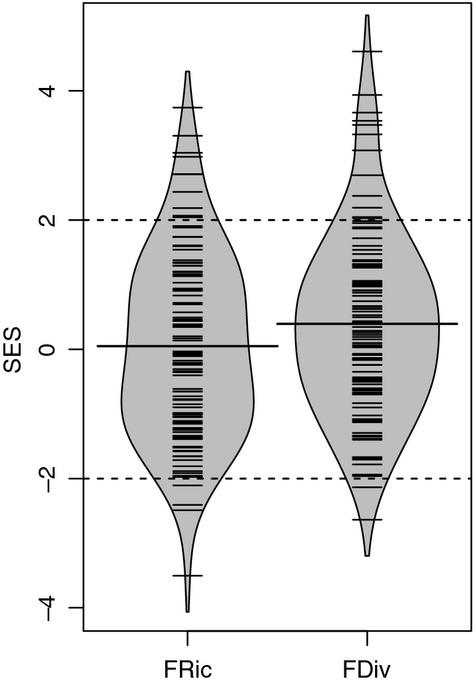
Bean plots showing the distribution of standardized effect size values (SES) for functional richness (FRic) and functional divergence (FDiv). Strips depict the individual observations (*n* = 113 communities) and thick lines indicate the mean. Density kernels were estimated on the basis of the individual SES values. Negative or positive SES values indicate that the trait diversity is lower or higher than expected. Strips outside the range of −2 to 2 indicate communities that have trait diversity values that are significantly different from those estimated from 999 random communities.

The mean SES of FRic was not significantly different from zero (Fig.1 and Table1), indicating that the multivariate range of dispersal and persistence traits within the grassland sites is, on average, not significantly higher or lower than expected from random. However, the values of FRic were highly variable across the landscape, and more grassland sites than expected contained communities with significantly lower (*n* = 9) or higher (*n* = 10) than expected FRic (Table1). Multivariate FRic was positively correlated with the range in trait values for longevity and epizoochory, whereas the ranges of endozoochory and wind dispersal potential were both negatively associated with FRic (Fig.3b and Table S2). In contrast to FRic, the mean SES of FDiv was, on average, higher than expected (Fig.1 and Table1), indicating a general tendency for species within the grassland sites to have higher levels of distinct/alternative dispersal and persistence strategies than expected from a random draw of species from the regional species pool. A higher than expected number of sites (*n* = 10) contained communities with a greater than expected FDiv, but only a few sites (*n* = 4) contained communities that had significantly lower than expected FDiv values. Multivariate FDiv was positively correlated with trait divergence in both seed bank persistence and adult plant longevity (Fig.3c, Table S2).

**Table 1:** Mean standardized effect sizes (SES) for functional richness (FRic) and functional divergence (FDiv; significance levels from one-sample *t-*tests) and the number of communities (*n* = 113 in total) that had FRic and FDiv values lower or higher than expected from 999 random communities (significance levels from one-tailed binomial test)

	FRic	FDiv
Mean SES	0.05 n.s.	0.39**
Lower than expected (*n*)	9**	4 n.s.
Higher than expected (*n*)	10***	10***

*** *P *≤* *0.001

** *P *≤* *0.01; n.s., non-significant.

### Relationships between Dispersal Trait Diversity and Local, Landscape and Historical Filters

The minimal adequate non-spatial models for both FRic and FDiv had spatially non-independent residuals (Table2). This residual autocorrelation was removed by the inclusion of pre-selected spatio-phylogenetic filters in the regression models.

**Table 2:** Minimal adequate regression models (GLMs) of the relationship between the standardized effect size of the dispersal trait diversity indices functional richness and functional divergence (FRic and FDiv) and the current and historical descriptors of the grassland communities and their surrounding landscape

	FRic		FDiv	
	Non-spatial	Spatio-phylo	Non-spatial	Spatio-phylo
Intercept	−0.168 n.s.	−0.109 n.s.	0.332**	0.332**
Grass.1938			0.475***	0.413**
Grass.2004			−0.402**	−0.248 n.s.
Age	0.234****	0.271*	0.293**	0.356**
Grazing			−0.353**	−0.188 n.s.
Tree.cov	−0.656***	−0.545***		
Tree.cov²	0.218*	0.158 n.s.		
Grazing × Grass.1938			0.457***	0.463***
Global Moran's *I*	0.023*	0.001 n.s.	0.024*	0.006 n.s.
Filters		P1		P3
AIC	381.35	356.05	374.7	364.6
*R*²adj	0.187	0.355	0.239	0.31

See ‘Materials and methods’ for variable abbreviations.

Non-spatial, non-spatial models; Spatio-phylo, models including spatially-structured phylogenetic filters (eigenvectors; see Fig. S2); Global Moran's *I*, Moran's *I* coefficient of autocorrelation; AIC, Akaike information criterion; *R*²adj, adjusted *R*².

*** *P* ≤ 0.001

** *P* ≤ 0.01

* *P* ≤ 0.05

**** *P* ≤ 0.1; n.s., non-significant.

**Fig 2:**
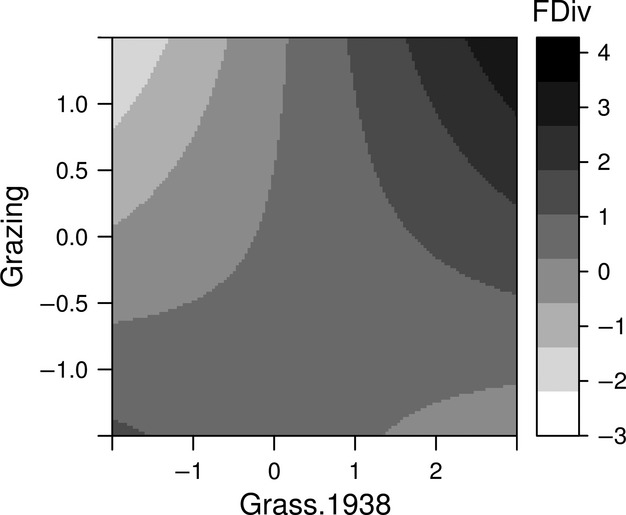
The interactive effect of present-day grazing intensity (Grazing) and the amount of grassland in the historical landscape (Grass.1938) on dispersal trait diversity (standardized effect size of functional divergence; light grey to black shading).

None of the dispersal trait diversity indices (SES of FRic and FDiv) were significantly explained by current landscape configurations (Grass.2004) or grassland area (Area). Instead, both FRic and FDiv were significantly positively associated with grassland age (Age, Table2), indicating that the multivariate range of dispersal and persistence traits that are occupied by the species, as well as the degree to which species within local communities have alternative/distinct dispersal and persistence strategies, is higher in older grassland sites that have been continuously grazed over long periods of time. FRic was also strongly negatively associated with the percentage cover of trees within the sites (Tree.cov), and FDiv showed a significant, positive association with the percentage of grassland habitat in the surrounding landscape in 1938 (Table2). However, FDiv was mainly explained by the interaction between the percentage of grassland in the surrounding historical landscape in 1938 and the current within-site grazing intensity (Grazing × Grass.1938; Table2, Fig.2). FDiv increased with grazing intensity, but only if the sites were surrounded by a large proportion of grassland habitat in the historical landscape: grassland communities in currently well-grazed sites that were also well connected in the past contain species that have high levels of distinct/alternative dispersal and persistence strategies.

## Discussion

Communities and their associated functions may be more resilient to environmental change if the species comprising the communities have the potential to disperse and persist by a wide range of different strategies (Mayfield, Ackerly & Daily 2006; Ozinga *et al.* 2009). But what factors determine the diversity of dispersal and persistence traits within local communities?

This study quantified the extent to which multivariate dispersal and persistence trait diversity in plant communities is explained by the current and historical characteristics of grassland sites and their surrounding landscape. Trait diversity was highest in sites that had a long continuity of grazing management and in sites that were surrounded by large amounts of grassland habitat in the historical landscape in 1938. Dispersal trait diversity also showed a positive relationship with current grazing intensity – but only in sites that were well connected in the historical landscape. Successful dispersal, involving multiple strategies, within the historical landscape, as well as the long-term availability of suitable microsites (gaps) for establishment within sites, is likely to have contributed to a high diversity of dispersal and persistence strategies within the present-day grassland sites.

### Null Model Analysis

To assess whether there are filtering processes that either constrain the diversity of dispersal or persistence traits or select for alternative dispersal and persistence strategies, observed trait diversity values need to be compared with the trait diversity values generated by a null model (Gotelli & Graves 1996). Because the null model used in our study maintained both levels of species richness within sites as well as species frequencies across the whole study landscape, the detection of higher or lower than expected trait diversity values provides a conservative indication of the presence of filtering processes (Gotelli & Entsminger 2003; Kembel & Hubbell 2006).

**Fig 3:**
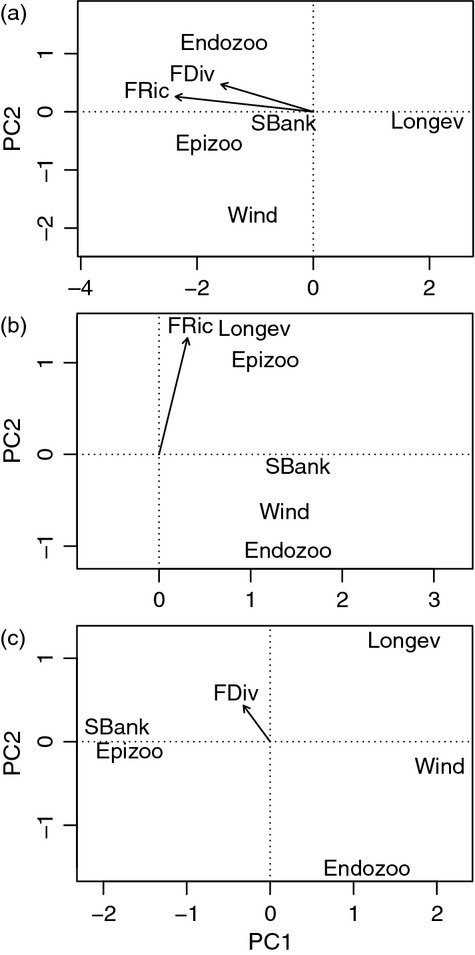
Biplots from principal components analyses illustrating the relationship between the within-site (*n* = 113) multivariate dispersal trait diversity (standardized effect size of functional richness and functional divergence) and (a) the site-level mean values, (b) the trait ranges and (c) the divergences for the five dispersal and persistence traits. Wind = wind dispersal potential; Epizoo = epizoochory; Endozoo = endozoochory; Longev = adult plant longevity; SBank = seed bank persistence. The directions of the arrows indicate positive correlations between multivariate trait diversity and the mean, range or divergence of the respective trait (see also Table S2).

The observed values of FRic were, on average, not significantly different from random expectations. The mean SES was close to zero (Table1 and Fig.1), indicating that there are no dominant filtering processes that either consistently constrain the multivariate range of dispersal and persistence traits or consistently select for species that differ in their dispersal and persistence strategies. However, the fact that more grassland sites than expected had significantly higher or lower FRic values than predicted from random communities (Table1) suggests that there are trait-based filtering processes whose relative importance varies with varying environmental conditions (e.g. management intensity, landscape complexity or history) within the study system (Kembel & Hubbell 2006; Pakeman, Lennon & Brooker 2011). Whereas the local habitat and landscape characteristics of some sites are likely to have acted as filters that restrict the multivariate range of dispersal and persistence traits, a different set of habitat characteristics in other sites may select for species with a wider range of (distinct) dispersal and persistence strategies. Lower than expected levels of trait diversity in some sites, combined with higher than expected trait diversity in other sites, appear to have resulted in average levels of trait diversity across the landscape that do not deviate from random predictions (Schamp & Aarssen 2009). The study by de Bello *et al*. (2011) (see also Vandewalle *et al.* 2013) points out that combining multiple traits into measures of functional diversity (as is the case with FRic or FDiv in our study) may make it difficult to assess (i) the extent to which a multivariate functional diversity index is driven by particular traits and/or (ii) whether opposing response directions in different traits contribute to low levels of multivariate functional diversity. The fact that, in our study, FRic was positively correlated with the range of trait values for both adult plant longevity and epizoochory potential (Fig.3b and Table S2) suggests that the multivariate range of dispersal traits was mainly driven by the range of trait values in these two traits.

In contrast to FRic, FDiv was, on average, higher than expected (Table1). The fact that more sites than expected (*n* = 10) had significantly greater than expected FDiv, whereas only four sites had a lower than expected FDiv, suggests that, across the grassland sites within our study system, there is a predominance of filtering processes that select for species with distinct/alternative dispersal and persistence strategies. However, our finding that the majority of sites showed patterns of multivariate FDiv that did not significantly differ from random expectations is likely to reflect the contrasting responses of FDiv in wind dispersal potential and FDiv in epizoochory potential or seed bank persistence (Fig.3c, Table S2), which may have resulted in low variation in FDiv values.

### Drivers of Dispersal Trait Diversity

The best model explaining the FDiv of dispersal and persistence traits (SES of FDiv) included descriptors of current and historical management regimes and landscape history. FDiv measures the extent to which between-species differences in dispersal and persistence traits are a reflection of extreme trait values. In our study, FDiv increased with both grassland age (Age) and the percentage of grassland habitat in the historical surrounding landscape in 1938 (Grass.1938; Table2), with values of multivariate FDiv being positively associated with FDiv in persistence traits and in epizoochory potential (Fig.3c, Table S2). A long continuity of grazing management in the old grasslands is likely to have ensured the long-term availability of gaps for establishment once seeds have arrived at a site, and may also allow for regeneration from the soil seed bank (Grubb 1977; Kahmen & Poschlod 2008). And the reserves of grassland habitat in the surrounding landscape represent the main dispersal source for the colonization of grassland fragments (Snäll *et al*. 2004). An earlier study in the same area (Purschke *et al.* 2012) showed that long-distance dispersal potential by wind and animals was explained by historical rather than by current landscape characteristics, and concluded that long-distance dispersal processes no longer contributed to the colonization of the remaining grassland fragments within the increasingly fragmented modern landscape. In the present study, communities with high FDiv values were associated with high mean values for long-distance dispersal potential by wind and animals (Fig.3a) as well as with high FDiv in adult plant longevity, seed bank persistence and epizoochory potential (Fig.3c and Table S2). The fact that FDiv was highest in the oldest sites (those with the longest grazing continuity), as well as in sites that were surrounded by large amounts of grassland habitat in the past, suggests that (i) the historical landscape structure has promoted dispersal by multiple vectors, and (ii) the presence of suitable microsites over long periods of time has ensured that these species could establish – generating communities that contain species that have a wide range of different dispersal and persistence strategies.

Although there was no direct association between FDiv and current grazing intensity, there was a highly significant interaction effect of present-day grazing intensity and the amount of grassland habitat in 1938 (Grazing × Grass.1938; Table2, Fig.2) for FDiv. Despite reduced levels of external recruitment in the modern landscape, grazing may allow the persistence of populations of long-distance dispersed species within grassland fragments – possibly because small-scale disturbance provides safe sites for continued internal recruitment (Purschke *et al.* 2012). But the results from the present study suggest that current grazing management will only be able to maintain a high diversity of dispersal and persistence strategies in sites that were surrounded by large amounts of grassland habitat in the historical landscape.

Functional richness, a measure of the multivariate range of dispersal and persistence traits, was lowest in young grasslands and in sites that are overgrown by trees. Whereas the low levels of FRic in the youngest grasslands are associated with a low proportion of long-distance dispersed grassland species (Fig.3a), the accumulation of litter and relatively high levels of shading within sites with high tree cover are likely to have selected for long-term persistent species (Fig.3a). An earlier study by Purschke *et al*. (2012), in the same study area, found that epizoochory potential increased with grassland age, but decreased with increasing tree cover, while adult plant longevity was negatively associated with grazing continuity and positively correlated with tree cover. The study concluded that, although the colonization of grassland species in present-day grasslands may be limited by long-distance dispersal at the landscape scale, species may persist at the local scale in open (less shaded) habitats. In the present study, the fact that high values in FRic were associated with (i) a high range in trait values for both adult plant longevity and epizoochorous dispersal potential (Fig.3b), and (ii) low levels of tree cover and a long grazing continuity (Table2) suggests that high levels of light availability in less shaded sites as well as the long-term availability of gaps for establishment are likely to have generated communities with a high range of alternative of long-distance dispersal and persistence strategies.

## Conclusions

Most studies of historical contingencies in the distribution of functional traits have focussed on biogeographic or evolutionary time-scales (Schimper 1898; Mooney & Dunn 1970; Moles *et al.* 2005; Swenson & Enquist 2007): historical anthropogenic impacts are often neglected (but see Knapp & Kühn 2012; Vandewalle *et al.* 2013). The results of the present study emphasize the importance of both history and current management regime (and their interactions) as determinants of multivariate dispersal and persistence trait diversity. Grazing continuity over long time periods enhances the diversity of different dispersal and persistence strategies within grassland communities. Trait diversity increases with current grazing intensity, but only in sites that were well connected to grassland areas in the past. The extent to which local grassland management strategies will be able to maintain a diversity of dispersal traits and buffer communities, and their associated functions, against future environmental changes is likely to depend on the historical context of sites within the landscape.

## Supporting Information

Figure S1 Phylogenetic tree of the 143 species in our study. **Figure S2.** Values of the spatio-phylogenetic filters (eigenvectors P1 and P3) mapped on the geographic x–y coordinates of the centroids of the studied grassland polygons. **Table S1.** Pearson's product-moment correlations (*r*) between the explanatory variables. **Table S2.** Pearson's product-moment correlations (*r*) between FRic and FDiv and the mean values, ranges and divergences for the five dispersal and persistence traits.
