# Supplementary material for: Interactive effects of landscape history and current management on dispersal trait diversity in grassland plant communities
Source: J Ecol. 2013 Dec 18;102(2):437–46. doi: 10.1111/1365-2745.12199 (PMC4258074; doi:10.1111/1365-2745.12199)
Supplement: Figure S1 — Phylogenetic tree of the 143 species in our study. Figure S2. Values of the spatio-phylogenetic filters (eigenvectors P1 and P3) mapped on the geographic x–y coordinates of the centroids of the studied grassland polygons. Table S1. Pearson's product-moment correlations (r) between the explanatory variables. Table S2. Pearson's product-moment correlations (r) between FRic and FDiv and the mean values, ranges and divergences for the five dispersal and persistence traits. [file jec0102-0437-SD1.doc]

**Supporting Information**

# **Table S1.** Pearson's product-moment correlations (r) between the seven explanatory variables. The significant correlation is indicated by bold text.

|  | Grass.1938 | Grass.1838 | Area | Tree.cov | Grazing | Age |
| --- | --- | --- | --- | --- | --- | --- |
| Grass.2004 | 0.25 | -0.10 | 0.16 | 0.04 | 0.16 | -0.06 |
| Grass.1938 |  | 0.19 | 0.02 | -0.05 | 0.14 | 0.25 |
| Grass.1838 |  |  | 0.03 | -0.09 | 0.23 | **0.30** |
| Area |  |  |  | 0.05 | 0.21 | 0.18 |
| Tree.cov |  |  |  |  | -0.25 | 0.16 |
| Grazing |  |  |  |  |  | 0.18 |

# **Table S2.** Pearson's product-moment correlations (r) between multivariate dispersal trait diversity (SES of FRic and FDiv) and the site-level mean values, ranges and divergence values of the five dispersal and persistence traits. Significant correlations are indicated by bold text.

|  |  |  |  | Mean | | | |  | Range | | | |  | Divergence | | | |
| --- | --- | --- | --- | --- | --- | --- | --- | --- | --- | --- | --- | --- | --- | --- | --- | --- | --- |
|  | FRic | FDiv |  | Epizoo | Endozoo | Longev | SBank |  | Epizoo | Endozoo | Longev | SBank |  | Epizoo | Endozoo | Longev | SBank |
| FDiv | 0.01 |  |  |  |  |  |  |  |  |  |  |  |  |  |  |  |  |
| Mean_Wind | **0.28** | -0.03 |  | **0.49** | 0 | **-0.35** | **0.27** |  |  |  |  |  |  |  |  |  |  |
| Mean_Epizoo | **0.21** | **0.37** |  |  | **0.42** | **-0.62** | **0.63** |  |  |  |  |  |  |  |  |  |  |
| Mean_Endozoo | **0.42** | 0.17 |  |  |  | **-0.60** | **0.60** |  |  |  |  |  |  |  |  |  |  |
| Mean_Longev | **-0.52** | **-0.23** |  |  |  |  | **-0.76** |  |  |  |  |  |  |  |  |  |  |
| Mean_SBank | **0.40** | **0.37** |  |  |  |  |  |  |  |  |  |  |  |  |  |  |  |
| Range_Wind | -0.04 |  |  |  |  |  |  |  | **0.26** | **0.30** | 0.07 | **0.19** |  |  |  |  |  |
| Range_Epizoo | **0.20** |  |  |  |  |  |  |  |  | 0.07 | **0.19** | 0.17 |  |  |  |  |  |
| Range_Endozoo | -0.11 |  |  |  |  |  |  |  |  |  | 0.1 | **0.35** |  |  |  |  |  |
| Range_Longev | **0.24** |  |  |  |  |  |  |  |  |  |  | **0.24** |  |  |  |  |  |
| Range_SBank | 0.02 |  |  |  |  |  |  |  |  |  |  |  |  |  |  |  |  |
| Divergence_Wind |  | -0.1 |  |  |  |  |  |  |  |  |  |  |  | **-0.76** | **0.51** | **0.32** | **-0.80** |
| Divergence_Epizoo |  | 0.15 |  |  |  |  |  |  |  |  |  |  |  |  | **-0.29** | **-0.38** | **0.74** |
| Divergence_Endozoo |  | 0.04 |  |  |  |  |  |  |  |  |  |  |  |  |  | 0.13 | **-0.25** |
| Divergence_Longev |  | **0.27** |  |  |  |  |  |  |  |  |  |  |  |  |  |  | **-0.34** |
| Divergence_SBank |  | **0.20** |  |  |  |  |  |  |  |  |  |  |  |  |  |  |  |

**Fig. S1.** Phylogenetic tree of the 143 species in our study, extracted from Durka & Michalski (2012).

**Fig. S2.** Spatio-phylogenetic filters (eigenvectors) describing phylogenetic variation between the studied grassland communities (i.e. spatio-phylogenetic autocorrelation) at larger (P1) and smaller (P3) spatial scales (see *Materials and Methods*). The values of the eigenvectors are mapped on the geographic x-y coordinates of the centroids of the studied grassland polygons. Large circles indicate high and small circles low values, respectively. Black circles correspond to negative and grey circles to positive values.

**References**

Durka W. & Michalski S.G. (2012) Daphne: a dated phylogeny of a large European flora for phylogenetically informed ecological analyses. *Ecology,* **93**, 2297-2297.
